# Intrasession and Between-Visit Variability of Sector Peripapillary Angioflow Vessel Density Values Measured with the Angiovue Optical Coherence Tomograph in Different Retinal Layers in Ocular Hypertension and Glaucoma

**DOI:** 10.1371/journal.pone.0161631

**Published:** 2016-08-18

**Authors:** Gábor Holló

**Affiliations:** Department of Ophthalmology, Semmelweis University, Budapest, Hungary; Universidade Federal do Rio de Janeiro, BRAZIL

## Abstract

**Purpose:**

To evaluate intrasession and between-visit reproducibility of sector peripapillary angioflow vessel-density (PAFD, %) values in the optic nerve head (ONH) and radial peripapillary capillaries (RPC) layers, respectively, and to analyze the influence of the corresponding sector retinal nerve fiber layer thickness (RNFLT) on the results.

**Methods:**

High quality images acquired with the Angiovue/RTVue-XR Avanti optical coherence tomograph (Optovue Inc., Fremont, USA) on 1 eye of 18 stable glaucoma and ocular hypertension patients were analyzed using the Optovue 2015.100.0.33 software version. Three images were acquired in one visit and 1 image 3 months later.

**Results:**

PAFD image quality for all images necessary to calculate reproducibility was sufficient to analysis only in 18 of the 83 participants (21.7%) who were successfully imaged for RNFLT. Intrasession coefficient of variation (CV) ranged between 2.30 and 3.89%, and 3.51 and 5.12% for the peripapillary sectors in the ONH and RPC layers, respectively. The corresponding between-visit CV values ranged between 3.05 and 4.26%, and 4.99 and 6.90%, respectively. Intrasession SD did not correlate with the corresponding RNFLT in any sector in either layer (P≥0.170). In the ONH layer sector PAFD values did not correlate with the corresponding RNFLT values (P≥0.100). In contrast, in the RPC layer a significant positive correlation between the corresponding sector PAFD and RNFLT values was found for all but one peripapillary sectors (Pearson-r range: 0.652 to 0.771, P≤0.0046).

**Conclusion:**

Though in several patients routine use of PAFD measurement may be limited by suboptimal image quality, in the successfully imaged cases (21.7% of the study eyes in the current investigation) reproducibility of sector PAFD values seems to be sufficient for clinical research. In stable patients intrasession variability explains most of the between-visit variability. Sector PAFD variability is independent from sector RNFLT, a marker of glaucoma severity. In the RPC layer sector PAFD and RNFLT show strong to very strong positive correlation.

## Introduction

**Fig 1:**
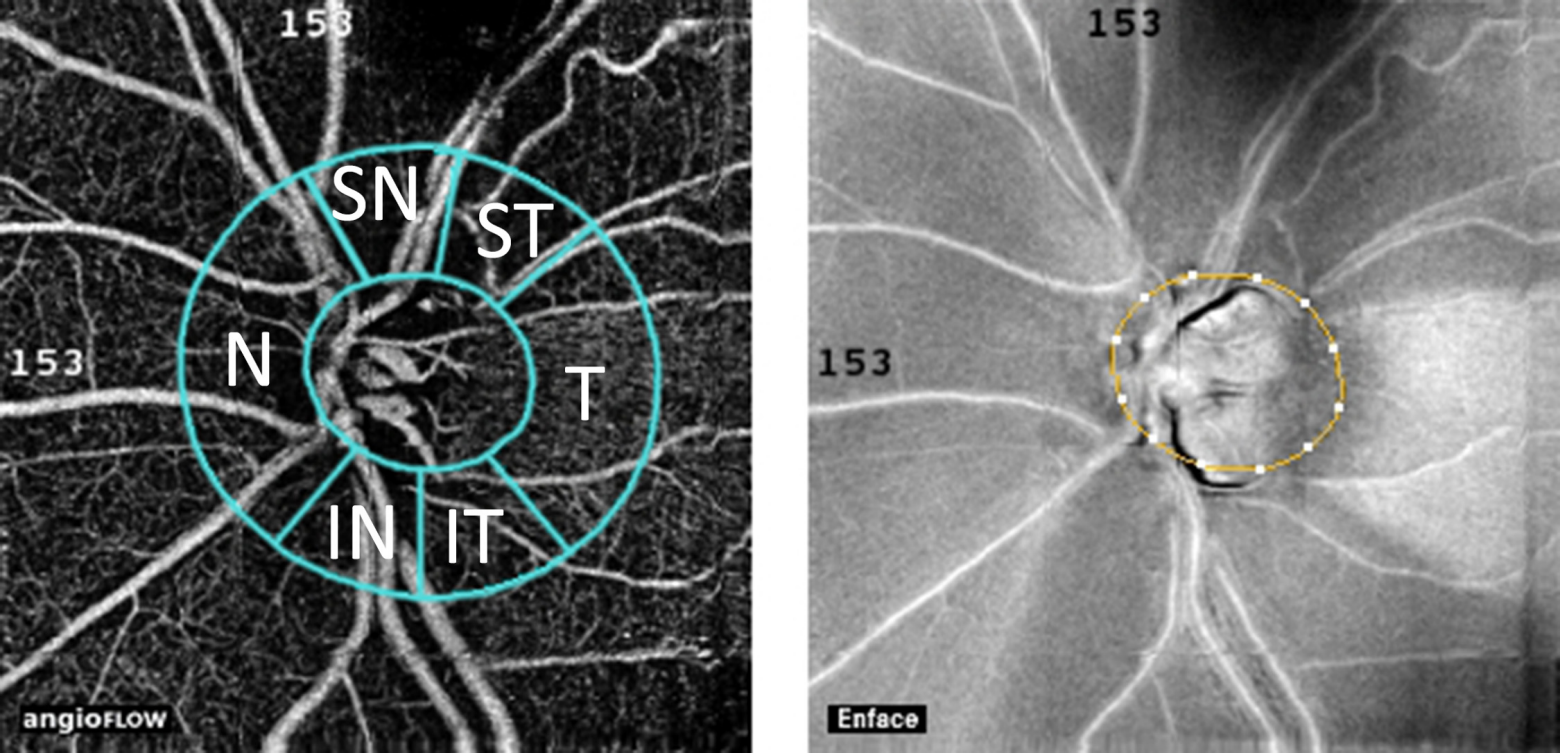
Positions of the peripapillary sectors given by the Optovue 2015.100.0.33 software version for peripapillary vessel density measurement with the Angiovue OCT.

Decreased ocular perfusion and vascular dysregulation in and around the optic nerve head have been considered as risk factors for the development and progression of open-angle glaucoma [1]. Therefore better understanding of the optic nerve head and peripapillary perfusion may provide clinically useful information for glaucoma research and care. The Angiovue optical coherence tomograph (OCT; Optovue Inc., Fremont, USA) angiography is a novel non-invasive technology, which was developed to measure vessel density and perfusion in various retinal layers in the macula, optic nerve head and peripapillary area, respectively [2–8]. Results of recently published investigations using this technology showed decreased disc and peripapillary vessel density in glaucoma, which was related to glaucomatous visual field deterioration and glaucoma stage [2, 3]. Before manufacturer-provided analysis software to disc and peripapillary measurements for Angiovue OCT angiography became available, research groups used their own software versions for analysis. The researcher-developed analyses did not evaluate the OCT angiography parameters for separate peripapillary sectors, but used the total peripapillary measurement area as one parameter. However, glaucomatous neuroretinal rim damage and decrease of retinal nerve fiber thickness (RNFLT) are frequently localized in early to moderate disease stages, thus separate analysis of the different peripapillary sectors may provide additional or more useful information compared to analysis of the total peripapillary area. Recently we have shown that peripapillary angioflow-density (PAFD; expressed in % of the measured area) measurement can identify decreased peripapillary perfusion early in the glaucomatous RNFLT thinning process, even prior to the development of clinically significant RNFL damage and visual field deterioration, and that decreased PAFD spatially corresponds with the damaged retinal nerve fiber bundles [8]. In the current investigation we evaluated a commercially not yet released software version (Optovue 2015.100.0.33 software version), which was developed for Angiovue OCT disc angiography to allow separate vessel density analysis in the Optic Nerve Head (ONH) layer (a layer spreading from the internal limiting membrane towards the vitreous body in 150 μm thickness) and the Radial Peripapillary Capillaries (RPC) layer (the layer between the outer limit of the retinal nerve fiber layer and the internal limiting membrane) for the disc area, the total peripapillary measurement area, and the temporal, superotemporal, superonasal, nasal, inferonasal, and inferotemporal peripapillary sectors (Fig 1), respectively. To evaluate the potential suitability of this analysis method for clinical research intrasession and between-visit reproducibility, the relationship between measurement variability and the spatially corresponding RNFLT, and the relationship between vessel density values and the spatially corresponding RNFLT values were analyzed for each layer and measurement area, respectively, in stable glaucoma and ocular hypertensive eyes.

## Materials and Methods

### Participants and Protocol

The research protocol was approved by the Institutional Review Board for Human Research of Semmelweis University, Budapest. Written informed consent was obtained from all participants before enrollment. All applicable institutional and governmental regulations concerning the ethical use of human volunteers were followed. All participants were white Europeans participating in a long-term imaging study in the Glaucoma Center of the Semmelweis University in Budapest. OCT angiography and retinal nerve fiber layer imaging were conducted prospectively between June and September 2015. In the first visit 3 OCT angiography measurements of the optic nerve head and peripapillary retina, and 1 retinal nerve fiber layer measurement were made. Three months later, in the second visit the eyes underwent 1 OCT angiography measurement. In both visits the participants underwent determination of the actual best corrected visual acuity, evaluation of the central 30-degree visual field using Octopus perimetry, and a detailed ophthalmological examination. Only visual field tests with less than 20% false positive and false negative responses, respectively, were used for classification. For retinal nerve fiber layer imaging only images with signal strength index (SSI) >50 were used.

The analyzed population comprised 10 under treatment ocular hypertensive eyes with normal optic nerve head and visual field (with mean defect [MD] less than 2 dB using Octopus perimetry normal strategy, Loss Variance [LV] less than 6 dB^2^, and no significantly decreased test point sensitivity value) and untreated intraocular pressure consistently above 21 mmHg; and 8 under treatment primary open-angle glaucoma eyes characterized with glaucomatous neuroretinal rim loss and reliable and reproducible visual field defect typical for glaucoma (inferior and/or superior paracentral or arcuate scotomas, nasal step, hemifield defect or generalized depression with Octopus perimetry MD higher than 2 dB using normal strategy). The demographics are shown in Table 1.

**Table 1:** Demographics of the study eyes.

Ocular hypertensive/ glaucoma eyes(n)	10 / 8
Age of the patients(years; median, quartiles)	60.048.0 69.0
Best corrected visual acuity(median, quartiles)	1.01.0 1.0
Spherical equivalent(Diopters; median, quartiles)	-0.5–2.5 0.0
Octopus perimetry MD*(dB; median, quartiles)	0.70.0 4.9
Number of eyes with Octopus MD	
-2.0 to 2.0 dB	10
2.0 to 6 dB	4
6.0 to 12.0 dB	1
12.0 to 19.0 dB	3

*In Octopus perimetry pathological mean deviation (MD) values are positive numbers

### Optical Coherence Tomography Angiography

The OCT angiography imaging of the optic nerve head and peripapillary retina, and the retinal nerve fiber layer measurement were made with the Angiovue/RTvue-XR Avanti OCT (Optovue Inc., Fremont, CA, USA). Only images with optimal image quality (SSI>50), no motion artifacts, vitreous floaters or other artifacts were selected for analysis. The detailed description of disc and peripapillary angioflow vessel density measurement technique has been published elsewhere [2–8]. In brief, the Angiovue OCT obtains amplitude decorrelation angiography images using an A-scan rate of 70,000 scans per second, a light source centered on 840 nm and a bandwidth of 50 nm. Each OCT-A volume contains 304 x 304 A-scans with two consecutive B-scans captured at each fixed position before proceeding to the next sampling location. Split-spectrum amplitude-decorrelation angiography is used to extract the OCT angiography information. Motion correction to minimize motion artifacts (vertical or horizontal lines, defocused, broken or shifted vessel and image segments) arising from microsaccades and fixation changes is used. Angiography information is displayed en face as the maximum of the decorrelation values within the corresponding layer. The technique of RNFLT measurements with the glaucoma protocol of the RTvue-XR OCT has been published earlier [8, 9].

### Determination of Peripapillary Angioflow-Density

One eye per participant (the eye with better image quality) was analyzed for PAFD measurement. We used a new and commercially not yet released software (the Optovue 2015.100.0.33 software version, Optovue Inc., Fremont, CA, USA) to measure PAFD (expressed in % of the measured area) in the disc area, the total peripapillary measurement area, and in each of the 6 peripapillary sectors (Fig 1) in 2 different layers, the ONH layer and the RPC layer, respectively. The software-provided peripapillary sectors are based on the Garway-Heath map [10]. The ONH layer is defined as the layer spreading from the internal limiting membrane towards the vitreous body in 150 μm thickness; and the RPC layer as the tissues between the outer limit of the retinal nerve fiber layer and the internal limiting membrane. Each layer corresponds with an en face structural image layer. The 4.5 mm x 4.5 mm scan size was used. The inner elliptical contour (which defines the optic nerve head) is obtained by automatic fitting an ellipse to the disc margin based on the OCT en face image. The peripapillary area is defined as the area between the inner and outer ellipses. The ring width between the inner and outer elliptical contour lines was 0.75 mm in all cases.

### Statistics

The STATA 6 software package was used for statistical analysis. Intrasession and between-visit reproducibility were characterized by the corresponding coefficient of variation (CV). Pearson's correlation was used to investigate the relationship between RNFLT and the corresponding vessel-density, and Spaerman’s correlation to investigate the relationship between RNFLT and the corresponding intrasession vessel-density SD values, respectively. P-values of less than 0.01 were considered to be statistically significant.

## Results

Eighty-three study participants were imaged during the 4 month of data collection, but sufficient image quality for all images necessary for the calculation of reproducibility was not achieved in 65 patients. The typical reasons of insufficient image quality were fixation losses, large eye movements, blinking due to dry eye related complaints, vitreous floaters and the combination of the above reasons. In the analyzed population (18 eyes, 21.7%) the mean ± SD PAFD values calculated for the disc area, the total peripapillary measurement area, and the 6 peripapillary sectors in the ONH and RPC layers, respectively, are shown in Table 2. For the disc area intrasession and between-visit CV were 3.05% and 6.53% in the ONH layer, and 3.38% and 6.46% in the RPC layer, respectively. The corresponding CV values found for the total peripapillary measurement area were 2.08% and 2.90%, and 2.48% and 3.84%, respectively (Tables 3 and 4). For the peripapillary sectors intrasession CV ranged between 2.30 and 3.89% in the ONH layer, and between 3.51 and 5.12% in the RPC layer (Tables 3 and 4). The corresponding between-visit CV values ranged between 3.05 and 4.26%, and 4.99 and 6.90%, respectively. Intrasession SD did not correlate with the corresponding RNFLT in any sector in either layer (P≥0.170). In the ONH layer sector PAFD values did not correlate with the corresponding RNFLT values (P≥0.100). In contrast, in the RPC layer a strong to very strong, statistically significant positive correlation between the corresponding PAFD and RNFLT values was found for all but one peripapillary sectors, and for the total peripapillary measurement area (Table 4).

**Table 2:** Peripapillary angioflow vessel-density (PAFD) in the optic nerve head (ONH) and radial peripapillary capillaries (RPC) layers, respectively.

	Disc area	Total peripapillary area	Peripapillary sectors					
			T	ST	SN	N	IN	IT
PAFD in the ONH layer (%, mean ± SD)	57.24 ± 6.4	62.07 ± 2.79	64.18 ± 2.84	63.55 ± 3.38	60.54 ± 3.13	60.05 ± 3.69	62.03 ± 4.75	63.16 ± 4.31
PAFD in the RPC layer (%, mean ± SD)	40.41 ± 5.3	55.43 ± 4.42	58.20 ± 4.43	50.01 ± 9.19	54.82 ± 5.45	53.54 ± 4.90	53.55 ± 6.58	56.43 ± 4.55

T, temporal; ST, superotemporal; SN, superonasal; N, nasal; IN, inferonasal; IT, inferotemporal; NA, not applicable

**Table 3:** Intrasession and between-visit coefficients of variation (CV), correlation coefficients (r) and their significance calculated for the relationship between intrasession standard deviation (SD) and the corresponding retinal nerve fiber layer thickness (RNFLT), and peripapillary angioflow vessel-density (PAFD) and the corresponding RNFLT, respectively, in the optic nerve head layer.

	Disc area	Total peripapillary area	Peripapillary sectors					
			T	ST	SN	N	IN	IT
Intrasession CV (%)	3.05	2.08	2.76	3.54	3.63	2.30	3.89	3.03
Between-visit CV (%)	6.53	2.90	3.22	3.66	4.21	4.13	4.26	3.05
r-value for correlation between intrasession SD and RNFLT*	NA	-0.112	-0.005	0.135	0.228	-0.217	-0.264	0.349
P-value for correlation between intrasession SD and RNFLT	NA	0.670	0.985	0.606	0.379	0.402	0.306	0.170
r-value for correlation between PAFD and RNFLT#	NA	-0.500	-0.242	0.019	-0.360	-0.219	-0.251	-0.413
P-value for correlation between PAFD and RNFLT	NA	0.041	0.349	0.942	0.156	0.397	0.331	0.100

T, temporal; ST, superotemporal; SN, superonasal; N, nasal; IN, inferonasal; IT, inferotemporal; NA, not applicable

*Spaerman’s correlation

# Pearson’s correlation

**Table 4:** Intrasession and between-visit coefficients of variation (CV), correlation coefficients (r) and their significance calculated for the relationship between intrasession standard deviation (SD) and the corresponding retinal nerve fiber layer thickness (RNFLT), and peripapillary angioflow vessel-density (PAFD) and the corresponding RNFLT, respectively, in the radial peripapillary capillaries layer.

	Disc area	Total peripapillary area	Peripapillary sectors					
			T	ST	SN	N	IN	IT
Intrasession CV (%)	3.38	2.48	3.51	5.12	3.87	3.69	4.76	3.72
Between-visit CV (%)	6.46	3.84	5.64	5.64	6.90	5.27	4.99	5.18
r-value for correlation between intrasession SD and RNFLT *	NA	-0.208	-0.215	-0.012	-0.254	-0.104	0.177	-0.204
P-value for correlation between intrasession SD and RNFLT	NA	0.422	0.407	0.963	0.326	0.690	0.497	0.432
r-value for correlation between PAFD and RNFLT#	NA	0.844	0.760	0.771	0.474	0.731	0.688	0.652
P-value for correlation between PAFD and RNFLT	NA	<0.0005	<0.0004	<0.0003	0.0550	0.0009	0.0022	0.0046

T, temporal; ST, superotemporal; SN, superonasal; N, nasal; IN, inferonasal; IT, inferotemporal; NA, not applicable

*Spaerman’s correlation

# Pearson’s correlation

## Discussion

In the current study we investigated intrasession and between-visit reproducibility of sector angioflow vessel-density values determined by a commercially not yet available software (the Optovue 2015.100.0.33 software version) developed to Angiovue OCT disc angiography by the OCT's manufacturer. This analysis software automatically provides PAFD values for the disc area, the total peripapillary measurement area and its 6 sectors in the ONH layer and the RPC layer, respectively. OCT angiography with the Angiovue OCT is a recently developed non-invasive method made for analysis of perfusion in the macula, and the optic disc and peripapillary retina, respectively [2–8]. Motion-corrected information reflecting on perfusion is automatically determined using split-spectrum amplitude decorrelation of subsequent images, and is presented for various retinal layers separately, parallel to the corresponding en face structural images. Reproducibility of PAFD determined for the total disc area and total peripapillary measurement area was recently investigated [2, 3]. The authors found favorable repeatability and a statistically significant relationship with glaucomatous visual field deterioration. However, they used their own software versions for image analysis and calculation, which did not provide data for peripapillary sectors separately. Since glaucomatous structural damage is frequently localized, separate analysis of the peripapillary sectors for PAFD may offer advantages over the use of the total peripapillary measurement area as a single parameter.

Our patient population comprised stable ocular hypertensive and primary open-angle glaucoma patients whose PAFD images were of high quality and free from any artifact. The severity of glaucomatous visual field deterioration ranged from no damage to severe damage, which allowed us to investigate the potential influence of sector RNFLT on the corresponding sector PAFD and its measurement variability. For the disc area the intrasession CV remained below 3.4%, and the between-visit CV below 6.6% in both layers. For the total peripapillary area the corresponding CV values were lower than 2.5% and 3.9%, respectively. These results are favorable and similar to the previously published corresponding data [2]. For the 6 peripapillary sectors, in the ONH layer intrasession and between-visit CV did not exceed 3.9 and 4.3%, respectively. In the RPC layer, where the retinal nerve fibers are located, the corresponding CV values did not exceed 5.2 and 6.9%, respectively. Intrasession variability explained most of the between-visit variability. These results suggest that sector PAFD results have a reproducibility which is sufficient for clinical research.

Intrasession SD of the PAFD measurements did not correlate with the spatially corresponding RNFLT in either layer, for any parameter. This suggests that measurement precision in the peripapillary sectors is not reduced in glaucoma compared to that in eyes with no structural and functional damage. Thus the probability of obtaining reproducible result does not depend on the severity of the disease, which is important for future research. In the RPC layer the spatially corresponding PAFD and RNFLT values showed a statistically significant, strong to very strong positive correlation in all but 1 sectors, and in the total peripapillary area. In contrast, in the ONH layer, where the retinal nerve fibers are not present, no correlation was found. These results mean that peripapillary PAFD in the RPC layer in fact shows RNFL-related vessel density, and suggest that image segmentation for PAFD measurement is satisfactory.

Our study has limitations. All our patients were white Europeans with low refractive error, thus limited conclusions can be drawn from our results with regard to other ethnic groups and eyes with high myopia or hyperopia. Of the 83 participants, who have all been successfully imaged for RNFLT and ganglion cell complex measurement with Optovue OCT for several years, only 18 had sufficient image quality in the total image area for all images necessary to calculate the reproducibility figures. We think that in addition to the high image quality requirement made to the current investigation the relatively long image acquisition time, the fixation problems common in advanced glaucoma, and the dry eye symptoms common in elderly glaucoma patients can be considered the main reasons of suboptimal image quality. This suggests that a considerable proportion of elderly glaucoma patients may not be optimal candidates for repeated OCT disc angioflow measurements. This may limit the routine clinical use of the technology, but with continued refinement may not significantly influence the use of PAFD measurements for research purposes. It is also important to note that while in the current investigation image quality had to be optimal in the total image area in all images for inclusion, in the studies published earlier no detailed definition of the required image quality was provided. This may explain the difference in the ratio of eligible and imaged eyes between previous studies and the current investigation [2–4].

In conclusion, in a selected stable ocular hypertensive and primary open-angle glaucoma population reproducibility of peripapillary sector PAFD determined with the Optovue 2015.100.0.33 software version using images obtained with the Angiovue OCT showed satisfactory short-term and long-term reproducibility. The measurement variability was independent from the spatially corresponding RNFLT. Peripapillary sector PAFD in the RPC layer showed strong to very strong positive correlation with the corresponding RNFLT values. Our results suggest that when image quality is optimal sector analysis of peripapillary vessel density may be reliably used for clinical research. However, it is important to note that high image quality in the total scan area in all images for an eye was obtained only in 21.7% of the participants in the current investigation, which suggests that further development of the technology is necessary.
