# Correction: Intrasession and Between-Visit Variability of Sector Peripapillary Angioflow Vessel Density Values Measured with the Angiovue Optical Coherence Tomograph in Different Retinal Layers in Ocular Hypertension and Glaucoma

**DOI:** 10.1371/journal.pone.0168231

**Published:** 2016-12-09

**Authors:** The PLOS ONE Staff

## Notice of Republication

This article was republished on August 25, 2016, to correct an error where an incorrect Fig 1 was published. The publisher apologizes for the error. Please download this article again to view the correct version.
